# Understanding Preeclampsia: Cardiovascular Pathophysiology, Histopathological Insights and Molecular Biomarkers

**DOI:** 10.3390/medsci13030154

**Published:** 2025-08-25

**Authors:** Kaltrina Kutllovci Hasani, Nurxhan Ajeti, Nandu Goswami

**Affiliations:** 1 Department of Obstetrics and Gynaecology, Medical University of Graz, 8036 Graz, Austria; 2 Department of Obstetrics and Gynaecology, Clinical Center Mother Theresa, 1000 Skopje, North Macedonia; nurxhanasani@gmail.com; 3 Gravitational Physiology and Aging Research Unit, Division of Physiology and Pathophysiology, Otto Löwi Research Center of Vascular Biology, Immunity and Inflammation, Medical University of Graz, 8036 Graz, Austria; 4 Centre for Space and Aviation Health, Mohammed Bin Rashid University of Medicine and Health Sciences, Dubai P.O. Box 505055, United Arab Emirates

**Keywords:** PE, endothelial dysfunction, placental pathology, cardiovascular risk, molecular biomarkers

## Abstract

Preeclampsia (PE) is not merely a pregnancy complication but a clinical manifestation of underlying vascular dysfunction with long-term health implications. It is diagnosed after 20 weeks of gestation as new-onset hypertension with proteinuria or organ involvement. The condition arises from impaired placental development, particularly defective spiral artery remodeling, which leads to placental ischemia and the release of antiangiogenic factors such as soluble fms-like tyrosine kinase-1 (sFlt-1) and soluble endoglin (sEng). These circulating factors contribute to systemic endothelial dysfunction, resulting in hypertension, inflammation, and multiorgan stress. Histopathological findings, including acute atherosis and abnormal vascular remodeling, further reflect the cardiovascular damage underlying PE. This review synthesizes emerging evidence on the vascular and histological mechanisms of PE, highlighting novel biomarkers such as microRNAs and neprilysin, and the potential of advanced diagnostic tools, including machine learning. Importantly, PE is now recognized not only as an obstetric disorder but also as an early marker of future cardiovascular disease. This paradigm shift emphasizes the need for personalized prevention strategies, close surveillance of high-risk women, and long-term cardiovascular follow-up. Pregnancy thus represents a critical window for early detection and intervention in women’s cardiovascular health.

## 1. Introduction

Preeclampsia (PE) is a life-threatening multisystem disorder of human pregnancy typically defined by new-onset hypertension after 20 weeks’ gestation accompanied by proteinuria or other maternal organ dysfunction [1]. It complicates ~2–8% [2] of pregnancies worldwide and remains a leading cause of maternal and perinatal morbidity and mortality [3]. Global analyses indicate hypertensive disorders (mainly PE and eclampsia) account for ~14% of maternal deaths, second only to hemorrhage [2,3]. Surviving PE grants elevated long-term risks, women have greater incidence of chronic hypertension, stroke, and cardiovascular disease (CVD), and offspring face higher risks of preterm birth, growth restriction, and later-life metabolic and cardiovascular disease [4,5,6]. These observations underscore that PE is not just an acute obstetric event but also a portentous precursor to future health implications.

Clinically, PE is diagnosed by maternal blood pressure ≥ 140/90 mmHg (on two occasions) after mid-pregnancy plus proteinuria (≥300 mg/24 h) or end-organ dysfunction (e.g., thrombocytopenia, elevated liver enzymes, renal insufficiency, cerebral disturbances) [1,4]. Severe features include BP ≥ 160/110, severe proteinuria, or significant organ injury (such as HELLP syndrome—hemolysis, elevated liver enzymes, low platelets) [6]. Early-onset PE (before 34 weeks) and late-onset PE (≥34 weeks) likely have differing pathogeneses (placental vs. maternal pre-dominant) [7,8]. Notably, definitive cure of PE requires delivery of the placenta; management hinges on balancing maternal severity with fetal maturity [9].

Over the past decade, research into PE pathophysiology has converged on a two-stage model: Stage 1 involves abnormal placentation with inadequate spiral artery remodeling, leading to placental ischemia while Stage 2 comprises the maternal syndrome triggered by placenta-derived factors causing systemic endothelial dysfunction and end-organ damage [10,11,12]. This narrative review synthesizes recent evidence on the cardiovascular changes in PE, histopathological placental lesions and emerging molecular biomarkers. We also review current approaches to predict and prevent PE, highlighting biomarkers like soluble fms-like tyrosine kinase-1 (sFlt-1) and placental growth factor (PlGF), and interventions such as low-dose aspirin. By understanding the intricate interplay between the placenta and maternal cardiovascular system in PE, we can better identify at-risk women and develop targeted therapies. Crucially, this review underscores how molecular dysregulation, particularly antiangiogenic signaling, oxidative stress, and immune activation, translates into systemic maternal organ dysfunction, manifesting as endothelial injury, cardiac remodeling, retinal vasospasm, and cerebral complications.

## 2. Results

### 2.1. Cardiovascular Pathophysiology of PE

#### 2.1.1. Abnormal Spiral Artery Remodeling

A defining placental defect in PE is the failure of normal remodeling of the uterine spiral arteries by invading trophoblasts [13]. In a healthy pregnancy, fetal extravillous trophoblasts invade the maternal decidua and transform the spiral arteries into high-caliber, low-resistance vessels, ensuring adequate uteroplacental blood flow [14,15]. In PE (especially early-onset forms), this physiological conversion is incomplete or shallow, leaving spiral arteries narrow, muscular, and hyper-responsive [16,17]. Histological studies show deficient trophoblast invasion and retention of the muscular and elastic components of spiral arteriole walls in PE placentas [16,17,18,19].

The result is uteroplacental hypoperfusion; the placenta is under-perfused and becomes ischemic during the second half of pregnancy. This placental ischemia is believed to trigger the release of various factors into the maternal circulation that drive the maternal syndrome of PE [16,17]. Notably, early-onset PE (EOPE, <34 weeks) is strongly associated with these placental implantation failures and often concomitant fetal growth restriction, whereas late-onset PE (≥34 weeks) may have near-normal spiral artery remodeling and is thought to result more from maternal constitutional factors or metabolic factors, such as pre-existing endothelial dysfunction, obesity, or diabetes [20,21]. While the early-onset and late-onset classification provides a clinically useful framework, recent evidence suggests these phenotypes lie on a spectrum rather than represent distinct pathologies. Late-onset PE can also exhibit placental lesions and features of underperfusion, whereas early-onset PE may present in women with metabolic risk factors. This overlap supports a continuum model of PE, acknowledging both shared and divergent pathophysiological pathways [7,19,20,22].

Clinically, PE is now recognized as not a single disorder but a syndrome of multiple phenotypes. Modern definitions, such as those of ACOG and ISSHP, no longer specify proteinuria in the criteria [1,4]. Instead, PE is defined by the onset of hypertension at or beyond 20 weeks’ gestation with either proteinuria or evidence of maternal organ dysfunction (e.g., liver or renal failure, thrombocytopenia, neurologic findings, or pulmonary edema) [1,4,5]. This broader model divides between traditional PE, non-proteinuric PE, chronic hypertension superimposed PE, and severe forms such as HELLP syndrome [5,6,7,8,9,10]. Of these, early- and late-onset PE are subtypes of particular interest, as they could differ in etiology, progression, and long-term maternal and fetal outcome [7,8,20].

Thus, impaired spiral artery remodeling sets the stage for placental ischemia and oxidative stress, the downstream effects of which are central to PE pathogenesis, particularly in the placenta-mediated, early-onset subtype, whereas late-onset PE reflects more of a maternal systemic inflammatory and vascular response, with a different underlying mechanism and placental morphology [13,16,17,18,19,20,21,22]. However, these differences should be interpreted within a broader spectrum of placental and maternal involvement, rather than strict dichotomy.

#### 2.1.2. Endothelial Dysfunction and Vascular Inflammation

The maternal syndrome of PE (Stage 2) is thought to be driven largely by widespread endothelial dysfunction secondary to the placental factors mentioned above [11,12]. Endothelial injury in PE manifests as reduced vasodilator production, increased vascular permeability, and a prothrombotic state [10]. A classic renal lesion in PE, glomerular endotheliosis, reflects this diffuse endothelial damage in the microvasculature [23]. Clinically, endothelial dysfunction translates to hypertension (from loss of vasodilators and heightened vasoconstrictors), proteinuria (due to glomerular leakage), and a tendency for clotting (due to endothelial activation and platelet aggregation) [23,24,25]. One hallmark is an imbalance of prostaglandins: PE placentas produce less prostacyclin (a vasodilator/platelet inhibitor) and more thromboxane A_2_ (a vasoconstrictor/platelet aggregator), favoring vasospasm and thrombosis [26]. Moreover, the maternal endothelium in PE is exposed to excess circulating anti-angiogenic factors like soluble Flt-1, which antagonize vascular endothelial growth factor (VEGF) and PlGF, further impairing endothelial health. Elevated levels of soluble endoglin(sEng), an anti-angiogenic protein that inhibits TGF-β signaling, have also been noted in PE, contributing to vascular dysfunction. Importantly, nitric oxide (NO) signaling is attenuated in PE [26,27]. Endothelial NO normally induces vasodilation; in PE, reduced NO bioavailability is observed, partly due to elevated levels of asymmetric dimethylarginine (ADMA)—an endogenous inhibitor of NO synthase [28]. A meta-analysis confirmed that circulating ADMA levels are significantly higher in women who develop PE compared to normotensive pregnancies. In 11 studies (total *n* = 1338), women who later developed PE exhibited an average standardized mean difference of +0.71 for circulating ADMA [29]. This suggests endothelial dysfunction precedes overt PE, and ADMA is implicated as both a marker and mediator. Notably, ADMA elevation was more pronounced after 20 weeks’ gestation, aligning with the timing of maternal syndrome onset [30,31].

#### 2.1.3. Arterial Stiffness: Another Key Feature of the Maternal Cardiovascular Maladaptation in PE

Healthy pregnancy is usually accompanied by reduced systemic vascular resistance and increased compliance [32,33]. In PE, chronic vasoconstriction and endothelial dysfunction lead to increased arterial stiffness, measurable as elevated pulse wave velocity (PWV) and augmentation index [34]. A meta-analysis of 23 studies found women with PE had significantly higher arterial stiffness indices than normotensive pregnant controls (pooled standardized mean difference ~1.6). Specifically, carotid-femoral PWV was ~1 m/s higher and augmentation index ~15 points higher in PE vs. normal pregnancy [35]. A more recent meta-analysis of 21 high-quality studies (n ~6500) found that PWV was elevated by ~0.67 m/s in women with PE [36], underscoring vascular stiffness as a consistent and quantifiable feature of PE-associated endothelial dysfunction. These changes are evident even when comparing PE to gestational hypertension, underscoring the unique vasculopathy of PE [34,37]. Increased stiffness not only contributes to hypertension in PE but may persist postpartum and predispose women to future CVD. Indeed, studies show arterial stiffness and subclinical atherosclerosis remain higher in women with prior PE long after delivery [35,38]. Endothelial function (often assessed by flow-mediated dilation, FMD) is also consistently impaired in women with PE both during pregnancy and in early postpartum [39]. A systematic review reported FMD responses were 1.7–12.2% lower in PE, and PWV responses were 13–26% higher, compared to unaffected pregnancies. These findings support the concept that PE mothers experience an accelerated cardiovascular aging or burden of risk factors [40].

#### 2.1.4. Retinal Vascular Alterations

Microvascular dysfunction is evident in PE; retinal imaging studies show that preeclamptic women have narrower retinal arteriolar diameters (lower central retinal artery equivalent, CRAE) and possibly narrower venules (CRVE) compared to normals. These retinal microvascular changes often precede clinical PE and may partially normalize after delivery, indicating a reversible microangiopathy tied to the hypertensive state [41,42].

The eye offers a noninvasive window to the microcirculation [43,44,45]. In PE, fundoscopic exams can show dynamic vasospasm (arteriolar narrowing), retinal hemorrhages, edema, or in severe cases signs of malignant hypertension (e.g., papilledema) [41,45]. A large prospective cohort (*n* ≈ 3391) with ~6 years postpartum follow-up showed that women with prior PE had significantly narrower retinal arteriolar calibers (adjusted difference ≈ −0.40 standard deviation score [SDS]; 95% CI −0.62 to −0.19) compared to normotensive women, while gestational hypertension also had milder narrowing (−0.20 SDS) but wider venules. These retinal microvascular changes persist long after pregnancy and may contribute to later cardiovascular disease risk [46]. A cross-sectional study found that pregnant women with hypertensive disorders had significantly smaller mean retinal arteriole diameters (~140 μm) versus normotensive pregnant women (~160 μm) [47]. Retinal venular caliber was also reduced. The degree of arteriolar narrowing correlated with higher blood pressure and lower fetal birth weight in PE, linking microvascular function to clinical severity [47,48]. Retinal arterioles and placental spiral arteries are both small resistance vessels, so this finding reinforces the concept of generalized microvascular dysfunction in PE [41,42,43]. Excitingly, recent research has leveraged retinal imaging for predictive modeling, using techniques like optical coherence tomography angiography (OCT-A) and even artificial intelligence to detect subtle vascular changes in early pregnancy [49,50]. One prospective study reported that changes in retinal microvascular caliber precede the clinical onset of PE, with first-trimester retinal arteriolar narrowing observed in those who later developed PE [48]. Additionally, a pilot using deep learning on fundus photographs could classify women at risk of PE with promising accuracy [48,50]. Although these tools are still investigational, they highlight the systemic nature of PE’s vascular impact [44,49]. Retinal metrics (CRAE, CRVE, and derived arteriole-to-venule ratio) could become part of multi-factor prediction models for PE in the future, complementing circulating biomarkers [44,48,49,50,51,52,53]. Clinically, the presence of visual disturbances in severe PE (blurred vision, scotomata) often reflects reversible retinal ischemia or cortical vision changes (PRES—posterior reversible encephalopathy syndrome) due to severely elevated blood pressure; these symptoms underscore the need for blood pressure control to prevent CNS and retinal complications [50,51,52]. However, these promising findings are largely based on small pilot studies, and few have undergone external validation [43,44,46,48,49,50,53]. Standardization of imaging protocols and prospective multi-center studies are needed to confirm their clinical utility.

#### 2.1.5. Hemodynamic Changes and Cardiac Function

PE is associated with distinctive maternal hemodynamic changes. While normal pregnancy is a high cardiac output, low-resistance state, preeclamptic women often show higher systemic vascular resistance and relatively lower cardiac output. Some studies suggest two phenotypes regarding hemodynamic changes: an early-onset “placental” PE with low cardiac output and high resistance, and a late-onset “maternal” PE with high cardiac output (often due to underlying metabolic syndrome). In both cases, blood pressure rises, but volume status may differ [1,2,4]. Women with PE tend to have an exaggerated vascular response to stress; for instance, they demonstrate more pronounced blood pressure increases with posture changes or exercise, reflecting a stiff, hyper-reactive circulation [21,25,26]. Maternal hemodynamic adaptation during pregnancy is crucial for cardiovascular stability. Studies have shown that women with inadequate adaptation, such as reduced plasma volume expansion or a blunted rise in cardiac output, are at higher risk of developing hypertensive complications. These findings support the potential of preclinical hemodynamic profiling as a tool to identify women at risk for PE and related disorders before clinical onset [21,24,25,53].

**Figure 1:**
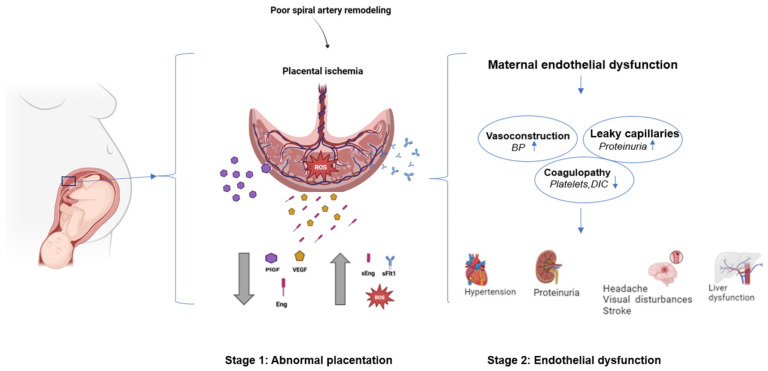
Pathophysiology of PE.

Cardiac function in PE can be affected; echocardiography often reveals diastolic dysfunction (impaired relaxation) and occasionally reduced systolic function in severe cases [53,54]. The combination of pressure overload and endothelial dysfunction can lead to subclinical cardiac strain [55]. Persistent cardiac remodeling postpartum has been documented in some PE survivors, which may contribute to the higher long-term heart failure risk [54,55]. Thus, PE can be viewed as a stress test for the maternal heart and vessels, unmasking latent endothelial and cardiac vulnerabilities [55,56,57]. Figure 1 illustrates the pathophysiology of PE.

This schematic illustrates the two-stage model of preeclampsia (PE) pathogenesis. In stage 1, abnormal placentation due to poor spiral artery remodeling (black arrow) leads to placental ischemia and oxidative stress from excess reactive oxygen species (ROS, red burst). This environment disrupts the angiogenic balance, with decreased pro-angiogenic factors (PlGF, purple hexagon; VEGF, yellow hexagon; downward gray arrows) and increased anti-angiogenic factors (sFlt-1, blue antibody; sEng, pink bar; upward gray arrows). In stage 2, these factors trigger maternal endothelial dysfunction characterized by vasoconstriction (increased BP ↑ leading to hypertension), increased vascular permeability (leaky capillaries resulting in proteinuria), and coagulopathy (platelet consumption and activation leading to disseminated intravascular coagulation, DIC). Clinically, this cascade manifests as hypertension, proteinuria, neurological symptoms such as headache, visual disturbances, and stroke, as well as hepatic dysfunction, reflecting the multisystem involvement of PE. Illustration created with BioRender.com. Data adapted from references [10,11,12].

#### 2.1.6. Systemic Vascular Injury Beyond the Cardiovascular System

PE is a multi-organ syndrome characterized by widespread vascular dysfunction, not confined to the heart and large vessels. Its systemic impact is most evident in maternal renal, hepatic, cerebral, and placental circulation, where endothelial injury and microvascular disturbances play a central role.

In the kidneys, the hallmark lesion is glomerular endotheliosis, in which endothelial cell swelling, loss of fenestrations, and narrowing of capillary lumens impair glomerular filtration, leading to proteinuria and rising serum creatinine [58,59]. This lesion is specific to PE and reflects direct endothelial damage and podocyte stress.

In the liver, PE can cause periportal hemorrhage, sinusoidal congestion, and hepatocellular necrosis, often reflected biochemically as elevated liver enzymes. In severe cases, HELLP syndrome (hemolysis, elevated liver enzymes, low platelets) may develop, indicating advanced microvascular injury and intravascular hemolysis [5,6].

Cerebral involvement may present as visual disturbances, seizures, or even stroke. One classic neuroimaging finding is posterior reversible encephalopathy syndrome (PRES), resulting from endothelial dysfunction and failure of cerebral autoregulation [60,61,62]. This complication reflects the breakdown of the blood–brain barrier, allowing fluid and protein leakage into the brain parenchyma.

Finally, the placenta itself is a site of intense vascular pathology, with features such as fibrinoid necrosis, infarctions, and acute atherosis of the spiral arteries [13,16,17,18,19]. These abnormalities contribute to chronic uteroplacental insufficiency, fetal growth restriction, and hypoxia-induced stress.

### 2.2. Histopathological Insights: Placental and Vascular Lesions

The placenta is the central organ in PE pathology. Histopathological examination of PE placentas consistently shows signs of malperfusion and vascular injury [13,16]. Key findings include the following.

#### 2.2.1. Inadequate Spiral Artery Remodeling and Acute Atherosis

As discussed, uterine spiral arteries in PE often remain narrow and muscular [18]. Pathologists note “failure of physiologic transformation” in the decidual segments of these arteries [16]. In normal placentation, by mid-pregnancy, trophoblasts replace the endothelium and muscular wall of these arteries with fibrinoid material [15]. In PE, many spiral arteries retain their endothelium and smooth muscle, indicating incomplete trophoblast invasion. This is quantified in studies showing a much lower proportion of remodeled vessels in PE vs. normals [16,17].

This lesion is so characteristic of hypertensive pregnancies that its presence in a placenta can retrospectively suggest undiagnosed PE. A large retrospective cohort study of >16,000 placentas, including nearly 1800 PE cases, found acute atherosis in ~10% of PE placentas vs. just 0.4% in normotensive pregnancies, and the lesion was strongly associated with early-onset disease, greater severity, and higher rates of fetal growth restriction [60]. Acute atherosis features fibrinoid necrosis of the vessel wall, foamy macrophages in the subintimal space, and lymphocytic infiltration [56,57]. Acute atherosis is found in the decidual arteries of a high proportion of PE placentas, especially in early-onset and severe cases [16]. It tends to occur in non-remodeled spiral arteries (i.e., that failed physiologic change) [17]. The depth and extent of acute atherosis correlate with PE severity and fetal growth restriction [56]. For instance, more deeply myometrial acute atherosis lesions are associated with earlier onset PE and worse outcomes [16]. These lesions likely reflect an immune-mediated attack on the vessel, possibly triggered by endothelial damage and lipid deposition [56,57]. Interestingly, they may also link to maternal future cardiovascular health: the presence of placental acute atherosis is postulated to contribute to the later CVD risk in PE mothers. It essentially mirrors atherosclerosis on a smaller scale and could indicate an underlying propensity to vascular inflammation [14,15,16].

#### 2.2.2. Villous Maldevelopment and Inflammatory Cell Infiltrates

PE placentas often show villous infarcts (areas of ischemic necrosis), syncytial knotting (exaggerated clustering of nuclei in trophoblast epithelium due to hypoxia), and distal villous hypoplasia (less branching of terminal villi) [8,9,10,11,12]. These changes indicate chronic ischemia [8]. Increased fibrin deposition in the intervillous space is also common [19]. In severe cases, gross placental lesions like infarcts and abruption (placental separation with hemorrhage) may be present, reflecting the extreme of placental vascular pathology [12,13,14,15,16,17].

Decidual biopsies in PE show enhanced inflammation—e.g., increased macrophages and T cells in the placental bed, and altered uterine natural killer (NK) cell populations (which play a role in normal spiral artery remodeling) [16]. Some studies have noted that an excessive maternal inflammatory response in the decidua may contribute to shallow invasion [57]. For example, high levels of Th17 cells and cytokines in decidua are reported in PE, pointing to an immunological component in failed tolerance of the fetoplacental unit [22,23,24,25]. While RCTs are lacking, mechanistic and immunologic studies support the involvement of immune dysregulation and chronic placental inflammation in the pathogenesis of PE.

#### 2.2.3. Glomerular Endotheliosis, Cardiac and Cerebral Changes

Although not a placental histology, it is worth mentioning the classic renal lesion unique to PE [58,59]. Electron microscopy of maternal kidneys reveals swollen, vacuolated glomerular endothelial cells and occlusion of capillary lumens by protein-rich material, so-called endotheliosis [58]. This lesion underlies the proteinuria in PE and usually resolves after delivery. Its presence can help confirm a diagnosis of PE on kidney biopsy and highlights the systemic nature of endothelial injury (as glomeruli are essentially specialized capillaries) [58,59,60].

In cases of eclampsia or fatal PE, autopsies have shown microscopic petechial hemorrhages and fibrin thrombi in multiple organs [61]. In the brain, hypertensive ruptures or ischemic lesions (stroke, hemorrhage) may occur, and some patients develop posterior reversible encephalopathy syndrome (PRES) with edema on imaging [62]. In the liver, periportal hemorrhages and fibrin deposits can occur (the lesion of HELLP syndrome). These severe manifestations, while not specific to PE (they result from extreme hypertension or DIC), underscore how PE’s pathology extends beyond the placenta into end organs via the circulation [4,5,6].

The histopathology of PE strongly supports the model of a placental-origin disease: the placenta shows the hallmarks of underperfusion and maternal maladaptation (failed remodeling, infarcts, atherosis) [12,16,17], and these placental failings instigate widespread maternal vascular damage (seen in kidneys, liver, brain) [5,6,59,62]. Examination of the placenta after delivery is therefore invaluable. A large clinicopathologic study found that among patients with PE, those whose placentas had acute atherosis and multiple infarcts had more severe hypertension and higher rates of fetal growth restriction [12,56]. Interestingly, not all cases of PE have overt placental histopathology; late-onset PE often has near-normal placental morphology, suggesting maternal constitutional factors can also lead to the syndrome despite a relatively healthy placenta [8,19]. This aligns with emerging concepts that “maternal PE” (often late onset) may result more from maternal cardiovascular dysfunction (e.g., endothelial susceptibility, obesity, chronic hypertension) than classic placental ischemia [19,26]. Nonetheless, even in these cases, the placenta usually shows at least subtle signs of malperfusion (like increased syncytial knots or slight infarction rates) if scrutinized [6,18].

### 2.3. Molecular and Cellular Mechanisms of PE

Unraveling the biological pathways from placental dysfunction to maternal disease: in Table 1, molecular and cellular mechanisms of PE are summarized, highlighting key pathways involved in angiogenic imbalance, oxidative stress, immune dysregulation, and endothelial dysfunction.

#### 2.3.1. Angiogenic Imbalance: A Central Pathway in Disease Progression

Over the last two decades, one of the greatest breakthroughs in PE research has been the discovery of disrupted angiogenic signaling [25,27]. In a healthy pregnancy, the placenta supports vascular development through the release of pro-angiogenic molecules, primarily vascular endothelial growth factor (VEGF) and placental growth factor (PlGF). These factors cause proper adequate endothelial function and promote vasodilation and angiogenesis [20,23,27].

In PE, however, this delicate balance is shattered. The ischemic placenta begins to release unusually high levels of soluble fms-like tyrosine kinase-1 (sFlt-1), a circulating decoy receptor that binds VEGF and PlGF, rendering them inactive. As a result, VEGF and PlGF become unavailable to maternal tissues, compromising endothelial repair and vascular tone. The end result is diffuse endothelial dysfunction, ranging from hypertension and proteinuria to end-organ ischemia [14,17,23,27].

Elevated sFlt-1 and suppressed PlGF can be identified well before symptoms develop. The ratio of sFlt-1 to PlGF has also appeared in the clinical setting as a method for ascertaining disease risk, with ratios > 38 suggesting an anti-angiogenic condition, and lower ratios offering high negative predictive value for exclusion of PE in the short term [25,27,28].

Another molecule, soluble endoglin (sEng), a shed form of the TGF-β co-receptor, rises in severe cases, particularly in PE associated with HELLP syndrome, amplifying endothelial injury by interfering with TGF-β-mediated vascular protection. The presence of both sFlt-1 and sEng intensifies vascular instability. While delivery remains the only curative therapy, new treatments, utilizing sFlt-1 apheresis and experimental treatments targeting this pathway, underscore its central role in pathogenesis [4,5,9,27,28].

#### 2.3.2. Oxidative Stress: Fueling Vascular Injury

The preeclamptic placenta is characterized by increased oxidative stress, and largely brought about by episodic perfusion and episodic ischemia followed by reperfusion. This generates reactive oxygen species (ROS) such as superoxide and peroxynitrite, which damage lipids, proteins, and DNA [25,28]. Studies consistently show elevated oxidative markers, like malondialdehyde and 3-nitrotyrosine, alongside reduced antioxidant enzyme levels (e.g., glutathione peroxidase, superoxide dismutase) [28]. A meta-analysis of 23 placental studies confirmed significantly increased oxidative damage (e.g., lipid peroxides, 8-OH-dG) and decreased antioxidant enzyme activity (SOD, GPx) in PE compared to normotensive pregnancies [63].

This redox imbalance diminishes nitric oxide (NO) availability, worsening vasoconstriction and endothelial dysfunction. Moreover, oxidative stress is not just a consequence but a driver of disease progression: it enhances placental shedding of anti-angiogenic factors like sFlt-1 and sEng, establishing a harmful feedback loop [26,28]. While antioxidant therapy with vitamins C and E has failed to prevent PE in clinical trials, newer agents such as melatonin and N-acetylcysteine are under active investigation [64]. However, the trials were relatively small and larger RCTs are required to confirm efficacy and safety. ADMA (asymmetric dimethylarginine), an endogenous inhibitor of NO synthesis, accumulates under oxidative conditions due to impaired degradation by DDAH enzymes. This further suppresses NO availability. L-arginine supplementation trials, a precursor to NO, have shown some benefit in high-risk females, suggesting that re-establishment of NO signaling may offer a therapeutic avenue [27,28,65].

#### 2.3.3. Immune Dysregulation: A Breakdown of Tolerance

PE represents a deviation from the immune tolerance that is present in pregnancy to a pro-inflammatory state. The immune cell populations in the decidua, primarily NK cells and macrophages, are deficient in their function and account for the reduction of trophoblast invasion. The predominance of M1 macrophages, coupled with a lack of tissue-remodeling M2 macrophages, creates a hostile environment for placental development [66].

At a systemic level, maternal immune dysregulation is manifest as elevated concentrations of pro-inflammatory cytokines (e.g., IL-6, TNF-α), elevated neutrophil activation, and production of neutrophil extracellular traps (NETs). In some cases, preeclamptic women develop autoantibodies against the angiotensin II type 1 receptor (AT1-AA), which have the capacity to activate vascular pathways independent of ligand binding, inducing vasoconstriction and amplifying release of sFlt-1 [66,67].

Of particular interest is the Th17/Treg axis. In healthy pregnancies, regulatory T cells (Tregs) enlarge to prevent fetal rejection. In PE, this balance is disrupted: Treg levels decline while Th17 cell producers of IL-17 increase, driving inflammation. This immune imbalance appears to be modulated in part by placenta-derived microRNAs, such as miR-210 and miR-155, which influence T cell differentiation and cytokine expression [68,69].

The complement system is also involved. Activation fragments are frequently found in PE placentas, and rare mutations in regulatory genes like CD46 or factor H have been linked to atypical presentations [69].

#### 2.3.4. Vascular Dysfunction: NO, ADMA, and Angiotensin Sensitivity

Apart from angiogenic breakdown and inflammation, the vascular endothelium itself is functionally and structurally remodeled in PE. eNOS, the enzyme involved in the synthesis of NO, is preferentially downregulated in placental and perhaps systemic vessels, resulting in prolonged vasoconstriction [24,26].

ADMA levels, as previously noted, are elevated, inhibiting NO synthesis and reinforcing endothelial dysfunction [32,33,34]. Concurrently, thromboxane synthase is elevated, and prostacyclin synthase reduced, tilting the balance of thromboxane–prostacyclin towards vasoconstriction and platelet aggregation [26].

The renin–angiotensin system is also deranged in PE. Even with decreased circulating levels of renin—presumably due to plasma volume contraction—there is enhanced sensitivity to angiotensin II. AT1 receptor autoantibodies further enhance this sensitivity by activating the receptor independent of angiotensin, constituting another level of vascular disease [15,24].

PE has an identifiable epigenetic signature in maternal and placental tissues. DNA methylation studies have demonstrated altered patterns of genes involved in angiogenesis, inflammation, and vasculature function. For instance, PGF and NOS3 gene hypermethylation has been linked to their reduced expression in PE placentas [13,23].

Transcriptomic analyses validate these findings. PE placentas exhibit increased expression of sFlt-1, leptin, and INHBA, and decreased levels of the most significant angiogenic transcripts. These are signs of a gene expression pattern that is under stress and inflamed, and disturbed trophoblast function [13,23,25].

MicroRNAs also adjust this network. Hypoxia-responsive miRNAs such as miR-210 and inflammatory miRNAs like miR-21 and miR-155 are consistently upregulated in PE and are thought to impair invasion and endothelial communication. These miRNAs, especially when encapsulated in exosomes, may alter gene expression in distant maternal tissues and have potential as circulating biomarkers [24,68,69].

#### 2.3.5. Neuroendocrine Stress: The Role of Cortisol

Another compelling layer of complexity comes from the neuroendocrine system. Chronic maternal stress and dysregulation of the HPA axis have been linked to PE. One of the biomarkers getting greater attention is hair cortisol concentration, which reflects chronic exposure to cortisol. Women who develop early-onset PE often have higher hair cortisol levels both preconception and at mid-pregnancy, suggesting chronic stress as a contributing factor [70]. A multicenter case–control study showed that women who developed early-onset PE had significantly higher hair cortisol concentrations (HCCs) in the preconception and second-trimester segments, indicating chronic elevated cortisol exposure well before clinical onset; these cortisol trajectories were notably steeper than in normotensive pregnancies [71]. Although there is no meta-analysis specifically comparing HCC in PE vs. control groups, a broader meta-analysis of 29 perinatal studies found only a weak, non-significant association between psychological distress and HCC, suggesting that HCC may reflect underlying pathophysiology beyond perceived stress alone [72].

Cortisol is harmful to trophoblast invasion, increase insulin resistance, and exacerbate endothelial dysfunction. Additionally, the placenta itself produces corticotropin-releasing hormone (CRH), which is elevated in PE and may contribute to vasospasm and preterm labor [70,73].

**Table 1:** Molecular and cellular mechanisms of PE.

Mechanism	Key Features	Key Molecules/Markers	Clinical Relevance	Reference No.
Angiogenic Imbalance	Disruption between pro- and anti-angiogenic factors	increased: sFlt-1, sEng decreased: PlGF,	sFlt-1/PlGF ratio > 38 is predictive of PE; sEng elevated in severe PE and HELLP	[5,25,27,28]
Oxidative Stress	Increased ROS from placental ischemia; impaired antioxidant defenses	increased: MDA, 3-nitrotyrosine, ADMA decreased: SOD, GPx	Promotes endothelial dysfunction and reduces nitric oxide availability; therapeutic target	[26,27,28]
Immune Dysregulation	Pro-inflammatory shift and breakdown of maternal–fetal tolerance.	increased: TNF-α, IL-6, NETs, AT1-AA, Th17, miR-210/miR-155 decreased: Tregs,	Leads to inflammation, poor placentation, and vascular dysfunction; potential for immunotherapy	[65,66,67,68,69]
Vascular Dysfunction	Endothelial injury and abnormal vasoregulation.	increased: ADMA, Thromboxane, AT1-AA decreased: eNOS, prostacyclin,	Results in vasoconstriction, hypertension, and platelet aggregation	[24,32,33,34]
Epigenetics and Transcriptomics	Changes in gene expression and DNA methylation in placental tissue	increased: Hypermethylation of PGF, NOS3; leptin, INHBA, sFlt-1; miR-210, miR-155	Molecular markers of placental stress and dysfunction; potential for early diagnostics	[23,25,68,69]

This table summarizes key molecular and cellular pathways implicated in the pathophysiology of preeclampsia (PE). Each mechanism is defined by characteristic biological features, associated molecular markers, and its clinical relevance. The pathways include angiogenic imbalance, oxidative stress, immune dysregulation, vascular dysfunction, epigenetic modifications, and neuroendocrine stress. For each, relevant biomarkers are listed alongside their diagnostic value, role in severity stratification, and potential as therapeutic targets. Data source: compiled from references [5,23,24,25,26,27,28,32,33,34,65,66,67,68,69].

### 2.4. Biomarkers and Molecular Diagnostics of PE

#### 2.4.1. Acknowledged Clinical Biomarkers: Angiogenic Factors

The imbalance between pro-angiogenic and anti-angiogenic factors plays a central role in the pathophysiology of PE (PE). The soluble fms-like tyrosine kinase-1 (sFlt-1)/placental growth factor (PlGF) ratio has been used as a standard biomarker for PE diagnosis and risk stratification [27,74,75]. The sFlt-1/PlGF ratio is a robust predictive biomarker: a meta-analysis of 15 studies (*n* ≈ 20,000) reported 80% sensitivity and 92% specificity for diagnosing PE, with excellent negative predictive value [74]. The ratio even before symptom onset helps in ruling in or out PE in suspected cases.

Clinically, a ratio of sFlt-1/PlGF ≤ 38 is practically excluding the development of PE within the subsequent 1–2 weeks with a negative predictive value as close to 99% [27]. Conversely, rising ratios correlate with rising severity and imminent onset, guiding clinical management, especially in the preterm context [74,75]. In Table 2, both established and emerging biomarkers of PE are presented, with emphasis on their diagnostic and prognostic utility.

#### 2.4.2. Promising Biomarkers: Expanding the Diagnostic Toolkit

Placental Protein 13 (PP13): produced by the syncytiotrophoblast, it plays a role in placental attachment and vascular remodeling [76]. Reduced first-trimester PP13 levels have been associated with higher risks for PE and placental dysfunction [77]. A meta-analysis assessing first- and early second-trimester biomarkers including placental protein 13 (PP13) revealed low predictive accuracy when used individually [78]. However, variability in assay methods and inconsistent performance across studies have limited its clinical application. Combining PP13 with other markers, such as PAPP-A and uterine artery Doppler, may enhance early detection, but standardization and validation problems persist [76,78].

Asymmetric Dimethylarginine (ADMA): an endogenous nitric oxide synthase inhibitor, which is elevated in women who develop PE, reflecting endothelial dysfunction. While promising, its predictive value is moderate compared to angiogenic markers. ADMA concentrations are linked with markers of oxidative stress and may be influenced by systemic inflammation, situating it within a broader vascular health profile [29,30,31]. A meta-analysis of 11 prospective comparisons (*n* ≈ 1338 women) found that early pregnancy circulating ADMA levels were significantly higher in those who later developed PE (standardized mean difference (SMD) ≈ 0.71, with greater elevation when measured at or after 20 weeks’ gestation (SMD ≈ 0.89) compared to measurements before 20 weeks (SMD ≈ 0.56) [29]. Another meta-analysis of 10 case–control studies (*n* ≈ 1129) confirmed that ADMA levels were markedly higher in PE versus controls, particularly in early-onset or severe disease (SMD ≈ 1.15 overall; rising to ≈2.41 in severe PE), however, despite this elevation, only a small number of studies have evaluated ADMA’s predictive accuracy, reporting moderate performance, with ROC AUCs around 0.76 [79,80].

Circulating MicroRNAs (miRNAs) have garnered attention as non-invasive biomarkers due to their stability in plasma and placenta-specific expression. A meta-analysis reported that panels of miRNAs, such as miR-210, miR-155, and miR-323, can distinguish PE cases from healthy pregnancies with good accuracy (AUC ~0.8). Exosomal miRNAs, packaged in placenta-derived extracellular vesicles, may offer greater diagnostic accuracy, reflecting the placental environment and maternal response. However, technical challenges in isolating and profiling exosomal content remain barriers to clinical translation [68,69,70].

Vitamin D: An Emerging Biomarker: recent research has highlighted the significance of vitamin D in pregnancy, particularly concerning its role in PE. Vitamin D deficiency is prevalent among pregnant women worldwide, with studies indicating that low levels of serum 25-hydroxyvitamin D [25(OH)D] are associated with an increased risk of developing PE [81,82]. A systematic review and meta-analysis encompassing 33 randomized controlled trials with over 10,000 participants demonstrated that vitamin D supplementation during pregnancy significantly reduced the risk of PE by approximately 45% [81]. Another large network meta-analysis of 27 randomized controlled trials involving over 28,000 women found that vitamin D supplementation alone or with calcium was associated with a reduced risk of preeclampsia, with pooled risk ratios ranging from 0.43 to 0.57, suggesting a potential preventive benefit that warrants further validation in large-scale trials [83]. Importantly, the association between vitamin D deficiency and PE risk is confounded by factors such as geographic location, supplementation practices, and baseline nutritional status. Results from intervention trials remain associative [81,83,84], suggesting that while deficiency may signal increased risk, causality is not established.

Metabolomics and Oxidative Stress Markers: PE is associated with systemic metabolic disturbances. Metabolomic profiling has revealed alterations in amino acid ratios, oxidative stress byproducts, and lipid metabolism [85]. Elevated uric acid and malondialdehyde (MDA) levels suggest oxidative injury but lack specificity [86,87]. Dyslipidemia, including elevated triglycerides and reduced HDL cholesterol, is also common in PE and contributes to endothelial dysfunction [88,89]. Although antioxidant supplementation trials have not yielded clinical benefits, these findings enhance understanding of PE pathophysiology and may aid in differentiating PE from other hypertensive or renal disorders [90,91].

Renal and Placental Markers (NGAL, sNEP): Neutrophil Gelatinase-Associated Lipocalin (NGAL) is also becoming an early biomarker for kidney injury, even in the context of PE (PE). The levels of urinary NGAL have been shown to increase in those women who develop PE later on, suggesting its potential use in detecting preclinical renal injury before clinical manifestations develop [92,93]. However, the sensitivity of NGAL as a standalone diagnostic test for acute kidney injury (AKI) secondary to PE is limited. While NGAL is raised in severe PE or eclampsia, it is modestly sensitive in differentiating PE with and without AKI [94,95]. Furthermore, raised levels of NGAL have been identified in women with PE post-delivery, which implies that renal tubular injury may persist after the period of delivery [96].

Soluble neprilysin (sNEP), which degrades vasoactive peptides, has been linked to better vascular function in high-risk pregnancies. Preliminary findings suggest that higher circulating sNEP may be protective by reducing vasoconstrictive peptides, though further research is needed to determine its utility as a biomarker or therapeutic target [53]. A recent study reported that placental expression of neprilysin is upregulated in PE and is associated with inflammatory markers, suggesting a link between sNEP, inflammation, and PE pathophysiology [97,98].

Detection Methodologies for PE Biomarkers: Commonly used techniques for measuring biomarkers in PE include enzyme-linked immunosorbent assays (ELISAs) for angiogenic factors (sFlt-1, PlGF), chemiluminescence immunoassays for PP13, liquid chromatography–mass spectrometry (LC-MS) for ADMA, and quantitative PCR (qPCR) for circulating and exosomal microRNAs [27,29,68,76]. NGAL and vitamin D levels are typically assessed via immunoturbidimetric or chemiluminescent assays. While these methods offer sensitivity and specificity, issues such as inter-laboratory variability, cost, and need for standardization remain barriers to widespread adoption.

Critical Appraisal of Emerging Biomarkers: While several biomarkers show promise for early prediction and pathophysiological insight into PE, many remain investigational and face substantial barriers to clinical translation. Limitations include heterogeneity in assay methods (e.g., for PP13, ADMA, and miRNAs), small or homogenous study populations, lack of external validation, and poor reproducibility across ethnic and geographic cohorts [29,30,31,32,68,69,70,76,78]. Additionally, some markers (e.g., NGAL, vitamin D, MDA) show only modest diagnostic performance and may be influenced by comorbid conditions such as obesity, renal dysfunction, or inflammation [83,86,87,93,94,95,96]. Thus, while these tools enhance understanding of PE pathogenesis, their integration into routine screening or diagnosis requires larger, multi-center prospective validation studies, standardized protocols, and cost–benefit analyses.

#### 2.4.3. Biophysical and Imaging Markers

Uterine Artery Doppler: Elevated pulsatility index and diastolic notching between 20 and 24 weeks suggest impaired placental perfusion and predict early-onset PE. This method is incorporated into first-trimester screening protocols [76,98,99,100].

Pulse Wave Velocity (PWV): An indicator of human artery stiffness, how stiff or not stiff the arteries are. Healthy arteries have a natural stretch and recoil with every heartbeat. Yet if the arteries harden, this elasticity is lost, and it is harder for the heart to push blood through [33,34]. It has been proved in research that PWV is raised in women who later develop PE [35,36]. Surprisingly, in research, this increase of arterial stiffness, in the form of elevated PWV, predominantly occurs prior to clinical presentation of PE. This would mean that PWV could be an early warning sign in women who are already at risk for the development of PE, and a window of opportunity to enhance surveillance and early intervention would be provided [32,35].

Retinal Imaging: retinal microvasculature mirrors systemic microcirculation. Optical coherence tomography angiography (OCT-A) and AI-based fundus analysis show promise in early detection of vascular changes in women who later develop PE. Though investigational, retinal imaging could become a non-invasive complement to biomarker-based screening [40,41,42,48,50].

Cardiac and Cerebral Imaging: Echocardiography may reveal subclinical diastolic dysfunction in PE [54,55], while cerebral imaging (MRI) can detect PRES or microhemorrhages, especially in severe cases. These findings underscore the multi-organ impact of PE [62].

Limitations of Imaging Tool: although biophysical and imaging modalities such as uterine artery Doppler, pulse wave velocity, and retinal OCT-A offer promising insights into maternal cardiovascular and placental health, several challenges persist [62,99,100]. Many studies are exploratory or underpowered, with limited generalizability due to small, single-center cohorts [48,50]. AI-based fundus analysis, in particular, has yet to be externally validated across diverse populations, and suffers from potential overfitting and lack of algorithm transparency. Additionally, logistical constraints such as operator dependency, imaging access, and standardization of measurement thresholds must be addressed before widespread clinical implementation is feasible.

**Table 2:** Promising and acknowledged biomarkers in PE.

Biomarker	Change in PE	Clinical/Pathophysiological Significance	Evidence (Reference No.)
sFlt-1/PlGF ratio	increased: sFlt-1, decreased: PlGF	Indicates anti-angiogenic imbalance and placental dysfunction; widely used in diagnosis.	[27,74,75]
ADMA (Asymmetric Dimethylarginine)	increased	Endogenous NOS inhibitor; impairs endothelial function via reduced nitric oxide.	[29,30,31]
PP13 (Placental Protein 13)	decreased	Marker of impaired trophoblast invasion and early placental development.	[76,77,78]
miRNAs (e.g., miR-210, miR-155)	increased/decreased (type-dependent)	Reflect hypoxia and immune dysregulation; miR-210 is hypoxia-associated.	[68,69,70]
NGAL (Neutrophil Gelatinase-Associated Lipocalin)	increased	Early marker of renal tubular stress in PE.	[92,93,94,95,96]
Neprilysin (sNEP)	increased	Degrades vasoactive peptides; may influence vascular tone in PE.	[53,97,98]
Inflammatory cytokines (TNF-α, IL-6)	increased	Reflect systemic inflammation; TNF-α induces anti-angiogenic factors.	[68,69,70]
Oxidative stress markers (MDA, 8-isoprostane)	increased	Oxidative stress markers linked to placental ischemia.	[86,87,88,89,90,91]
Uric acid	increased	Associated with renal dysfunction and oxidative stress.	[86,87]
Vitamin D [25(OH)D]	decreased	Deficiency may impair angiogenesis and immune regulation.	[79,80,81,82]
PAPP-A (Pregnancy-Associated Plasma Protein A)	decreased	Early screening marker; linked to placental dysfunction.	[101,102,103]
Retinal microvasculature (CRAE/CRVE)	increased CRAE decreased CRVE	Non-invasive indicator of systemic microvascular dysfunction.	[40,41,42,48,50]
Pulse wave velocity (PWV)	increased	Indicates increased arterial stiffness before clinical PE onset.	[33,34,35,36]

This table summarizes both established and emerging biomarkers relevant to the diagnosis, risk stratification, and pathophysiological understanding of preeclampsia (PE). Biomarkers are organized according to their biological function and observed alterations in preeclamptic pregnancies. Specifically, they are grouped based on biological relevance into categories such as angiogenic imbalance (e.g., sFlt-1/PlGF), oxidative stress, renal and vascular dysfunction, immune activation, epigenetic regulation (e.g., miRNAs), and endothelial injury. This classification reflects the multifactorial nature of PE and facilitates a clearer understanding of its underlying mechanisms. Each entry outlines the direction of change in PE, clinical relevance, and supporting evidence derived from meta-analyses, clinical studies, and translational research. This structured overview enhances readability and serves as a concise reference for both established and investigational biomarkers in the context of PE. Data source: compiled from references [27,29,30,31,33,34,35,36,40,41,42,48,50,53,68,69,70,74,75,76,77,78,79,80,81,82,84,85,90,91,92,93,94,95,96,97,98,99,100,101,102,103].

### 2.5. Risk Factors and High-Risk Populations in PE

PE may happen in any pregnancy, but some women have elevated baseline risk. It is important that these high-risk groups be identified in order to provide early management, individualized treatment, and focused preventive interventions like aspirin prophylaxis [4,5,6,99,100,101,102,103].

#### 2.5.1. Established Clinical Risk Factors

Epidemiological studies and large-scale meta-analyses have clarified the key clinical risk factors that predispose to PE. Many of these factors reflect underlying placental insufficiency, endothelial dysfunction, or immunologic maladaptation [5,6,25,26]. Table 3 outlines the major maternal, obstetric, and genetic risk factors associated with the development of PE.

History of PE: A prior history of PE remains the strongest known predictor of recurrence. The relative risk increases up to 8–9-fold, especially if the previous case was early-onset or severe. This risk can raise to nearly 30–50% after two prior episodes, calling attention to vigilant monitoring in subsequent pregnancies [4,5,6].

Chronic Hypertension and Renal Disease: Women with established hypertension have approximately a 5-fold increased risk for superimposed PE [104,105]. These patients are more likely to have vascular and endothelial dysfunction predisposing them to poor placental adaptation. Chronic kidney disease also contributes to this, often occurring in association with hypertension [6,24,92].

Diabetes Mellitus: Both type 1 and type 2 diabetes significantly raise PE risk (RR ~3–4), likely as a result of chronic metabolic and endothelial stress. Gestational diabetes is also associated with a modestly increased risk, but the impact is smaller than for pregestational diabetes [1,6,105].

Autoimmune Disorders (e.g., APS, SLE): Antiphospholipid syndrome is a well-established risk factor, with PE in up to 17% of affected pregnancies [106]. Lupus and other autoimmune diseases (particularly those with vascular or renal involvement) also confer risk via chronic inflammation, endothelial damage, and prothrombotic state [1,6].

Nulliparity and New Paternity: PE is largely a disorder of first pregnancies, and nulliparous women carry approximately double the risk of multiparous women. This is accountable through failure of prior immune adaptation to paternal antigens. Of interest, a partner change between pregnancies re-sets this risk, emphasizing the role of immunologic priming [1,5,6,25,26].

Obesity: A high body mass index (BMI > 30) is one of the most modifiable risk factors, with a 2–3-fold increased risk of PE. The risk rises in a dose-dependent manner with increasing BMI [107]. Obesity contributes to systemic inflammation, oxidative stress, and insulin resistance, all factors known to impair placental development and vascular function [1,4,5,6,107].

Multiple Gestation: Twin or higher-order pregnancies confer a 2–3× increased risk of PE [108]. The presence of multiple placentas or a larger placental mass increases maternal vascular load and angiogenic factor demand, predisposing to endothelial dysfunction [1,5,6,25,26].

Advanced Maternal Age (AMA): Women over 35, especially those over 40, face increased PE risk [109], likely due to vascular aging, subclinical cardiovascular disease, and higher rates of comorbidities. At the other extreme, teenage pregnancies (<18 years) may also confer slight risk due to biological immaturity or socioeconomic disadvantage [1,4,5,6].

Family History and Genetic Predisposition: A maternal or sibling history of PE doubles the risk, highlighting the potential role of genetic factors such as FLT1 gene variants. This has led to growing interest in polygenic risk scoring in the future [1,5,103].

Assisted Reproduction and IVF: Pregnancies conceived via in vitro fertilization, particularly with donor oocytes, exhibit higher PE risk. The immune system may respond more aggressively to fully allogenic fetuses, contributing to abnormal placentation [1,4,5,6,110].

**Table 3:** Risk factors for PE.

Risk Factor	Relative Risk (RR)/Effect Estimate	Comment	Reference No.
History of PE	RR 8–9×	Recurrence rate up to 30–50% after two prior episodes	[4,5,6]
Chronic Hypertension	RR ~5×	Established independent risk factor	[6,24,90,104]
Chronic Kidney Disease	Not quantified	Consistently associated with increased risk	[6,24,92]
Type 1 and 2 Diabetes Mellitus	RR ~3–4×	Pre-existing DM carries higher risk than GDM	[1,6,105]
Gestational Diabetes	Modest increase	Risk lower than in pregestational DM	[1,6,105]
Autoimmune Disorders (e.g., APS, SLE)	Up to 17% incidence	Especially high in antiphospholipid syndrome	[1,6,106]
Nulliparity	RR ~2×	Baseline risk for first pregnancy	[1,5,6,25,26]
New Paternity	RR ~2×	Risk comparable to first pregnancy with new partner	[1,5,6,25,26]
Obesity (BMI > 30)	RR ~2–3×	Risk increases proportionally with BMI	[1,4,5,6,107]
Multiple Gestation	RR ~2–3×	Dose-dependent with number of fetuses	[1,5,6,25,26,108]
Advanced Maternal Age (>35, esp. >40)	Increased (not quantified)	Possibly related to endothelial aging	[1,4,5,6,109]
Family History (mother/sibling)	RR ~2×	Suggests genetic and shared environmental factor	[1,5,105]
Assisted Reproduction (IVF, donor egg)	Elevated risk (not quantified)	Possibly due to immune and placental factors	[1,4,5,6,110]
Ethnicity (e.g., Black women)	Increased (qualitative)	Even after adjusting for comorbidities	[1,4,5,6]
Low Socioeconomic Status	Increased (qualitative)	May reflect access to care, chronic stress	[1,4,5,6]
Psychosocial Stress	Increased (qualitative)	Elevated hair cortisol linked with early PE	[1,5,6,25]
Smoking During Pregnancy	Complex/conflicting	Not protective; not recommended	[111,112,113,114,115]

This table summarizes maternal, obstetric, and socioeconomic risk factors associated with an increased likelihood of developing preeclampsia (PE). Approximate effect estimates—such as relative risk (RR) or odds ratio (OR)—are provided where available, based on epidemiological studies and clinical guidelines. Risk factors are grouped into three categories: medical history (e.g., chronic hypertension, diabetes), pregnancy-related variables (e.g., nulliparity, multiple gestation, assisted reproduction), and demographic or psychosocial contributors (e.g., age, socioeconomic status, stress). Some factors, such as smoking during pregnancy, are included due to their complex epidemiological associations but are not recommended clinically due to adverse fetal effects. Abbreviations: RR—Relative Risk; OR—Odds Ratio; PE—Preeclampsia; GDM—Gestational Diabetes Mellitus; DM—Diabetes Mellitus; Qualitative—Risk association observed in studies but without a specific effect estimate. Data Source: compiled from references [1,4,5,6,24,25,26,90,104,105,106,107,108,109,110,111,112,113,114,115].

#### 2.5.2. Additional Risk Modifiers

Ethnicity and Socioeconomic Status: Black American women have increased rates of PE even after adjustment for comorbidities, likely a reflection of the interplay between genetic predisposition, chronic stress, and unequal care. Low socioeconomic status is additionally linked to risk, at least partly a reflection of dietary inadequacies and inadequate prenatal care [1,4,5,6].

Psychosocial Stress: Elevated maternal stress, measurable using biomarkers like hair cortisol, has been associated with an increased risk of PE. Chronic stress may potentially impair vascular and immune adaptations required in a healthy pregnancy [1,5,6,25].

Smoking: The so-called ‘smoking paradox’ refers to a counterintuitive observation from several epidemiological studies showing that maternal smoking during pregnancy is associated with a reduced risk of PE, with reported relative risk reductions of 20–50% [111,112,113]. A meta-analysis of 17 prospective studies encompassing over 1.8 million pregnancies found a significant inverse association between smoking during pregnancy and preeclampsia risk (RR = 0.67; 95% CI: 0.60–0.75), though the high heterogeneity and potential confounding warrant cautious interpretation and further large-scale validation [112]. This association has led to hypotheses involving nicotine-induced vasodilation, altered angiogenic balance (e.g., increased PlGF or decreased sFlt-1), or placental adaptation mechanisms. A dose–response trend has even been described, with heavier smoking linked to lower PE incidence, though this remains controversial. Quitting smoking in the first trimester did not appear to modify this effect in some analyses [112,113]. Despite these findings, no biologically robust, mechanistic explanation has been confirmed.

Although some epidemiological data show a lower incidence of PE among smokers, this so-called ‘smoking paradox’ must be interpreted with extreme caution [111,112,113,114,115]. The association is observational, subject to residual confounding and bias, and should not be construed as causal. Most critically, smoking is unequivocally harmful in pregnancy—it is associated with fetal growth restriction, placental abruption, preterm birth, stillbirth, and long-term offspring morbidity [114,115]. Therefore, its mention here serves only to illustrate the complexity of PE epidemiology, not to suggest any protective effect or clinical recommendation. Smoking cessation remains an essential component of prenatal care.

#### 2.5.3. High-Risk Stratification in Clinical Guidelines

Given the serious maternal and fetal complications associated with PE (PE), early identification of women at risk is a major goal in obstetric care [1]. Accurate prediction allows clinicians to initiate preventive measures such as aspirin and to tailor monitoring strategies to reduce adverse outcomes [1,4]. Existing preventative measures have progressed from clinical history and currently include biophysical measurement, biochemical markers, and even artificial intelligence [1,49].

Recent guidelines, including ACOG and ISSHP, recommend the identification of women with established high-risk factors like prior PE, chronic hypertension, diabetes, autoimmune disease, multifetal gestation, nulliparity, or two or more moderate-risk features like obesity or family history [1,4]. However, relying on clinical history alone has limited sensitivity: a significant number of PE cases still occur in women without obvious risk factors [4].

First-Trimester Combined Screening: One of the most practically used strategies is first-trimester combined screening, originally proposed by the UK’s Fetal Medicine Foundation (FMF) [100]. This algorithm, performed between 11 and 14 weeks of gestation, combines maternal risk factors along with mean arterial pressure (MAP), uterine artery Doppler parameters, and serum markers such as placental growth factor (PlGF) and pregnancy-associated plasma protein-A (PAPP-A) [76,99,102].

ASPRE was the trial that helped prove this method, showing that it is possible to predict 75% of early-onset PE cases with a 10% false-positive rate [102]. In this system, uterine artery Doppler is at the core: elevated pulsatility index or diastolic notch signifies impaired placental perfusion [76].

When these findings are combined with low PlGF levels, the ability to predict early PE improves significantly [74,76,99,102]. Women identified as high risk (e.g., an estimated risk > 1 in 100) may then be offered prophylactic interventions such as low-dose aspirin, which has been shown to reduce the incidence of early-onset PE [1,101,102].

Mid- to Late-Pregnancy Biomarkers: With increasing gestation, biochemical screening is especially useful in distinguishing evolving PE from nonspecific or other hypertensive syndromes [1,5]. Among such tests, the sFlt-1/PlGF ratio test stands out. It is especially useful in the second or third trimester when women present with increased blood pressure, headache, or gestational suspicion of intrauterine growth restriction but are yet to meet criteria for PE [27,74].

Low sFlt-1/PlGF ratio or normal PlGF value highly predicts that PE is unlikely to happen in the next 1–2 weeks and hence more conservative management is justified [27]. A meta-analysis of 15 studies (534 PE cases, 19,587 controls) showed that the sFlt-1/PlGF ratio predicts preeclampsia with 80% sensitivity and 92% specificity, supporting its value as a screening tool for both high- and low-risk pregnancies [74]. National health systems such as the UK’s NICE have incorporated PlGF testing in routine hospital practice for suspecting PE, indicative of earlier diagnosis and improved outcomes [105].

As pregnancy enters the second trimester, attention also turns to markers of placental insufficiency. Fetal growth restriction (FGR) and abnormal uterine artery Doppler findings at mid-pregnancy (around 20 weeks) may signal the onset of PE-related placental dysfunction [8,99]. They usually precede the clinical onset of PE and warrant increased fetal surveillance and prophylactic therapy consideration if not done already [6,7,8].

#### 2.5.4. Integration of Machine Learning and Digital Tools in PE Risk Prediction

Single-marker preeclampsia (PE) screening is certain to have its limitations, and this has led to the development of multi-marker prediction models. The best-established example is that of the ASPRE trial, in which it was demonstrated that maternal factors augmented by mean arterial pressure (MAP), uterine artery Doppler, placental growth factor (PlGF), and PAPP-A can detect over 90% of early-onset PE at a 10% false-positive rate. The algorithm has already been incorporated into routine antenatal care in some healthcare systems [101,102].

According to this, AI and ML are increasingly being researched as tools to complement PE risk stratification and individualize care. PIERS and fullPIERS algorithms apply clinical symptoms, blood pressure profile, laboratory results (i.e., creatinine, ALT, platelets), and angiogenic biomarkers (i.e., sFlt-1/PlGF) to predict severe maternal outcomes [50,103]. Parallel to this, ML algorithms have also been developed to add features such as uterine artery pulsatility index and retinal microvascular changes to gain a better understanding of cardiovascular and placental health [48,103].

Beyond hospital-based algorithms, platforms of telemedicine and home blood pressure monitoring are being combined with ML algorithms to spot trends in high-risk women. For example, electronic health tool-assisted analysis of electronic health records like home-measured weight and blood pressure as well as laboratory value trends for the development of early PE recognition [1,44]. A recent systematic review reported that ML algorithms incorporating clinical and biochemical parameters had AUC values ranging between 0.86 and 0.97, but their utility for early screening was suggested [44].

Nonetheless, these tools remain largely investigational. Most AI/ML models have been developed on small, relatively homogeneous collections and not externally validated in diverse populations. Algorithmic bias, overfitting, limited transparency (“black box” models), and a lack of integration into the clinical work flows are still significant barriers to general use [48,50]. Therefore, while this review mentions in passing the promise of AI to predict PE, such technologies at this juncture ought to be considered experimental adjuncts, not substitutes for proved clinical models. Large-scale, prospective, multi-center verification will be required to determine their eventual worth and safety in practice.

### 2.6. Prevention Strategies for PE

Despite delivery of the placenta being the only definitive cure for PE, modern prevention efforts aim to delay or avoid the onset of the syndrome, especially in women at elevated risk. These strategies target known pathophysiological mechanisms such as impaired placentation and endothelial dysfunction [4,5,6].

#### 2.6.1. Low-Dose Aspirin Prophylaxis

Low-dose aspirin (75–150 mg daily, typically 81 mg in the U.S.) forms the cornerstone of current preventive strategies for PE. Its mechanism involves irreversible inhibition of cyclooxygenase-1 (COX-1), which suppresses thromboxane A2 synthesis, a potent vasoconstrictor and platelet aggregator, while exerting relatively less inhibition on prostacyclin, a vasodilator and inhibitor of platelet aggregation. This selective action shifts the hemostatic balance toward vasodilation and antithrombotic activity, improving placental perfusion [1,4,5,6,102].

The landmark ASPRE trial demonstrated that administering 150 mg of aspirin daily from 11–14 weeks until 36 weeks of gestation reduced the risk of preterm PE by up to 62% in high-risk women identified through combined screening [102]. Complementing these findings, the ASPIRIN trial, a multinational, double-blind RCT involving nearly 12,000 nulliparous women with singleton pregnancies, showed that daily low-dose aspirin (81 mg), initiated between 6 and 13 + 6 weeks of gestation, significantly reduced the incidence of preterm birth (<37 weeks) with additional reductions in perinatal mortality and early preterm delivery before 34 weeks [116].

Based on this robust evidence, clinical guidelines from ACOG and ISSHP recommend initiating low-dose aspirin between 12 and 16 weeks of gestation in women with at least one high-risk factor (e.g., previous PE, chronic hypertension, diabetes, renal disease, autoimmune conditions) or two or more moderate-risk factors (e.g., obesity, nulliparity, family history of PE, advanced maternal age) [1,4,101,102].

Aspirin prophylaxis is widely regarded as safe, inexpensive, and well-tolerated. Recent evidence suggests that higher doses (100–150 mg) are more effective than lower doses (e.g., 60 mg) traditionally used in earlier trials. The therapy is typically continued until 36–37 weeks of gestation, although real-world adherence and individual risk factors (e.g., bleeding tendency) may affect clinical outcomes [101,102].

Calcium Supplementation: In populations with low dietary calcium intake, supplementation during pregnancy (1.5–2 g/day) has been shown to halve the risk of preeclampsia (PE), likely by reducing vascular smooth muscle excitability, improving endothelial function, and lowering blood pressure [117,118,119]. A Cochrane overview of 30 randomized controlled trials (RCTs) involving approximately 20,445 women reported a 49% relative risk reduction in PE (RR ≈ 0.51, 95% CI 0.40–0.66) and a 30% lower risk of gestational hypertension (RR ≈ 0.70) among women receiving calcium supplements compared to placebo [120]. Similarly, a meta-analysis that included 30 trials (N = 20,445) and a network meta-analysis (NMA) of 25 trials (N = 15,038) assessing calcium dosage confirmed that both low-dose (<1 g/day) and high-dose (≥1 g/day) calcium regimens are comparably effective in women with low baseline calcium intake, with no significant difference in efficacy between dosing strategies [121]. Supporting this, the WHO randomized trial, conducted in populations with habitual calcium intake below 600 mg/day, demonstrated that daily supplementation with 1.5 g calcium significantly reduced severe hypertension, eclampsia, adverse maternal outcomes (e.g., severe PE complication index; RR ~0.76), and neonatal mortality [122]. Consequently, calcium supplementation is specifically recommended by the WHO for pregnant women in regions with low dietary calcium intake, while it is not universally advised in populations with adequate calcium consumption [117].

Vitamin D: modulates immune responses, promotes a balance between pro-inflammatory and anti-inflammatory cytokines, and influences the expression of genes involved in angiogenesis and endothelial function, all critical in the pathophysiology of PE. These findings suggest that monitoring and managing vitamin D status could be a valuable component in the prevention and management strategies for PE [81,84]. A pooled analysis of 27 RCTs (4777 pregnant women) showed that vitamin D supplementation significantly reduced the risk of preeclampsia, especially when started before 20 weeks’ gestation, with higher doses linked to greater risk reduction. Similarly, a meta-analysis of 33 RCTs (10,613 participants) confirmed that vitamin D lowered the risk of preeclampsia by about 45% and preterm labor by 30%, although its impact on neonatal outcomes like low birth weight and Apgar scores was not statistically significant [82,83]. Thus, while low vitamin D levels may indicate increased risk, current evidence is insufficient to recommend routine supplementation for PE prevention in all populations.

#### 2.6.2. Lifestyle and Behavioral Interventions

Lifestyle interventions are universally recommended during pregnancy, although their effect on preeclampsia (PE) risk appears modest. A systematic review and meta-analysis of 23 randomized controlled trials involving 7236 overweight or obese pregnant women found that diet and/or exercise significantly reduced gestational weight but did not reduce the risk of PE (RR 1.01; 95% CI 0.80–1.27) or hypertensive disorders overall (RR 0.87; 95% CI 0.70–1.06) [123]. A more recent 2024 meta-analysis focusing on physical activity demonstrated 19% lower odds of gestational hypertensive disorders among exercisers (OR 0.81; 95% CI 0.65–1.00), with more pronounced benefits for yoga and mixed aerobic/anaerobic programs (e.g., yoga OR 0.28; 95% CI 0.13–0.58) [124]. Individual RCTs have further shown that structured exercise programs such as cycling for at least 30 min, three times per week can reduce PE frequency and improve maternal outcomes [125].

Restricting excessive weight gain, following a healthy diet, and engaging in regular physical activity are therefore sensible, particularly given the strong link between obesity and PE. While general salt restriction is not routinely advised, limiting high sodium intake is considered prudent [1,5,6]. Smoking cessation is also strongly recommended. Despite a paradoxical epidemiologic association suggesting a lower incidence of PE among smokers, systematic reviews and meta-analyses confirm that this is not a causal protective effect. The multiple, well-established risks of smoking during pregnancy including fetal growth restriction and placental complications far outweigh any speculative benefits [111,112,113].

Heparin, Statins, and Other Pharmacologic Strategies: In women with antiphospholipid syndrome, low-dose aspirin combined with prophylactic heparin is the standard of care to improve outcomes and reduce PE risk [1,5]. A meta-analysis of 14 RCTs (*n* = 1966) showed that adding low-molecular-weight heparin (LMWH) to low-dose aspirin significantly reduced the risk of preeclampsia (RR 0.59; 95% CI 0.44–0.79), SGA, gestational hypertension, and neonatal death without increasing bleeding risk [126]. Outside of confirmed thrombophilic disorders, heparin is not routinely recommended for PE prevention [1,101,102].

Emerging interest surrounds agents like metformin and statins. Metformin, used in diabetes and PCOS, may improve endothelial function and reduce oxidative stress. Preliminary studies suggest potential benefits in reducing gestational hypertension; robust data on PE are still pending [127].

Statins (notably pravastatin) have pleiotropic effects including improving endothelial function and increasing PlGF. Early studies and case series suggest benefit in high-risk women (e.g., prior early-onset severe PE), but statins remain experimental due to limited safety data in pregnancy. The NIH StAmP trial is currently investigating pravastatin for prevention in high-risk groups [128,129,130].

Timing of Delivery and Cardiovascular Follow-Up in PE: Close surveillance of high-risk women is essential to prevent severe complications. Regular prenatal visits, early baseline labs, and frequent monitoring for rising blood pressure or proteinuria help detect PE early. If diagnosed, timely delivery can prevent progression to life-threatening conditions such as eclampsia, stroke, or hepatic rupture. Current practice is to deliver women with PE without severe features at 37 weeks, and those with severe PE at or after 34 weeks—or earlier if clinical deterioration occurs [1,4,5,6]. Prophylactic magnesium sulfate for seizure prevention and antihypertensive therapy have significantly improved maternal outcomes in PE [4,5,6].

PE is now recognized as a marker for future cardiovascular disease. Guidelines recommend postpartum follow-up to monitor blood pressure, glucose, and lipid profiles. Women with prior PE should receive lifestyle counseling and cardiovascular risk assessment within 3–6 months postpartum and annually thereafter [1,4].

This “life-course” approach acknowledges that PE may serve as an early indicator of chronic conditions such as hypertension and coronary artery disease. Timely intervention may reduce long-term morbidity [1,4,5,6].

## 3. Discussion

PE is increasingly recognized as a complex cardiovascular syndrome with its origins during pregnancy but long-term consequences for maternal health [4,5,6]. Its pathophysiology involves intricate interplay between dysregulated placental development, systemic endothelial dysfunction, immune dysregulation, and cardiovascular maladaptation [4,5,10,11,13,22,24]. This multi-layered pathogenesis calls for a multidimensional strategy integrating clinical, biochemical, histological, and imaging-based findings [5,6,13,27]. While several epidemiologic studies show that women with a history of PE have increased cardiovascular risk [1,4,37], it is important to emphasize that these associations do not establish a direct causal relationship. Most of the available data are observational in nature, and residual confounding, including shared risk factors such as obesity, hypertension, and endothelial dysfunction, may contribute to both conditions. Therefore, caution must be taken not to infer causality from association alone.

One of the significant initiating mechanisms, particularly in early-onset PE, is the poor remodeling of the spiral arteries and consequent chronic uteroplacental ischemia [10,15,16,17]. The ischemic placenta releases anti-angiogenic proteins such as sFlt-1 and pro-inflammatory cytokines into the maternal circulation, causing global endothelial injury [5,24,26,27]. Biochemically, it appears as elevated ADMA levels, reduced bioavailability of nitric oxide (NO), oxidative stress, and an imbalance of vasoactive prostanoids [28,29,30,31]. These changes lead to clinical manifestations of hypertension, proteinuria, increased arterial stiffness, and vascular hyperreactivity [5,32,33,34].

Cardiovascular adaptation during uncomplicated pregnancy is characterized by reduced systemic vascular resistance and increased cardiac output [5,6].

In PE, however, this adaptive pattern is disturbed [5,6,20,21]. Hemodynamic study proves that the affected women exhibit increased systemic vascular resistance and, as a function of the phenotype, reduced or increased cardiac output [20,21]. Such various cardiovascular profiles, comparing placental-mediated early-onset PE with maternal-metabolic late-onset forms highlight the heterogeneity of the condition [7,19,20]. While the traditional division into placental and maternal subtypes of PE is useful, it may oversimplify the complex nature of the disease. Many cases present with overlapping features, so risk assessment should reflect this continuum [7,19].

Microvascular alterations, particularly in the retina, present an accessible and unique window to systemic vascular impairment [40,41,42]. Constriction of retinal arterioles and venules, observed both preterminally and with clinical symptomatology of PE, mirrors placental microangiopathy and is indicative of generalized endothelial impairment [41,42,43,44]. These alterations are often reversible postpartum but may serve as potential early risk markers in susceptible groups [42,44,47].

Histologically, PE placenta presents homogeneous evidence of maternal vascular malperfusion as infarcts, fibrinoid necrosis, syncytial knots, and acute atherosis [5,10,17,49,50]. Concurrently, glomerular endotheliosis of maternal kidneys and immune cell infiltration in the decidua present robust evidence of systemic inflammation and immune activation [5,46,53,93,94]. These mark the way PE is at the intersection of vascular, metabolic, and immunological dysfunction [5,11,25,53].

Several maternal risk factors predispose to PE. These include pre-existing hypertension and diabetes mellitus, obesity, chronic kidney disease, autoimmune disorders (e.g., systemic lupus erythematosus and antiphospholipid syndrome), advanced age, and assisted reproductive technique [5,6,55,56,57,58,59,60,61]. Numerous large-scale cohort and meta-analytic studies have shown that women with a history of PE have a significantly higher risk of developing chronic hypertension, ischemic heart disease, heart failure, and stroke later in life [4,37,54,55,83]. However, it is important to interpret these findings as associations, not established causal relationships. Shared risk factors such as endothelial dysfunction, obesity, insulin resistance, and genetic predisposition may contribute to both PE and subsequent cardiovascular disease [4,37]. Moreover, many of the existing studies are observational and cannot account for all confounders. Therefore, while PE represents a valuable early warning marker of cardiovascular vulnerability, further prospective, mechanistic, and interventional research is needed to determine whether and how PE contributes causally to future CVD [6,37,83].

Prevention has been variably successful. Low-dose aspirin (initiated before 16 weeks of pregnancy) is now prescribed in high-risk patients, and evidence indicates that it prevents early-onset PE from developing [99,100]. Calcium supplement therapy, especially in low-income individuals with low dietary intake, and lifestyle modifications such as weight maintenance, exercise, and smoking cessation also prevent risk [65,66,67,68,113,114,119,120,121,122,123,124]. However, they must be tailored and integrated into a broader model of pre- and post-pregnancy cardiovascular prevention [37,39,66,69].

Increasingly, PE is no longer regarded as an isolated obstetric event but as a sentinel risk factor for later cardiovascular disease [37,53,57]. Women with a history of PE have a high long-term risk of chronic hypertension, ischemic heart disease, heart failure, stroke, and renal impairment [58,59,104,107]. This appreciation reclassifies PE as an earlier window of opportunity for earlier detection and risk stratification of women at risk for long-term cardiovascular morbidity [101,102,103,104].

Future Directions: Refining early prediction models that utilize biochemical markers (e.g., sFlt-1/PlGF, ADMA), hemodynamic measurements, and retinal imaging, perhaps supplemented with artificial intelligence, in the future would be crucial [27,28,40,47,73,74]. Enhanced molecular profiling (e.g., proteomics, metabolomics, epigenetics) may unveil new targets for therapy and clarify dichotomous PE phenotypes [5,25,75]. Further focus on immunological mechanisms and maternal–fetal tolerance would enable better understanding of pathogenesis and timing of intervention [13,22,76,77]. Individualized prevention interventions and systematized postpartum cardiovascular monitoring must be prioritized, particularly in high-risk subjects [6,71,78].

Longitudinal cohort studies and randomized controlled trials are required not only to assess the long-term benefits of early intervention but also to better understand whether PE causally contributes to cardiovascular disease or serves primarily as an early marker of risk [70,71,79].

## 4. Materials and Methods

### 4.1. Literature Search Strategy

This narrative review was conducted to synthesize current evidence on the cardiovascular pathophysiology, histopathological features, and molecular biomarkers of PE (PE). We performed a structured literature search covering the period from 2015 to 2025, focusing on peer-reviewed publications in English. We initially retrieved 356 records through database searches. After removing 76 duplicates, 280 titles and abstracts were screened. Of these, 146 full-text articles were assessed for eligibility, and 120 were included in the final synthesis. Only peer-reviewed articles were considered. No formal risk-of-bias tool was applied, but systematic reviews, randomized trials, and large cohort studies were prioritized. The search was conducted across multiple biomedical databases including PubMed (MEDLINE), Embase, Web of Science, and Scopus. Search terms included combinations of keywords and Medical Subject Headings (MeSHs) related to PE (e.g., “PE”, “hypertensive disorders of pregnancy”) and to each of the core topics:

- Cardiovascular mechanisms (e.g., “endothelial dysfunction”, “arterial stiffness”, “hemodynamic”, “heart disease”),
- Placental and histopathological features (e.g., “spiral arteries”, “malperfusion”, “acute atherosis”),
- Molecular biomarkers (e.g., “sFlt-1”, “PlGF”, “microRNA”, “metabolomics”, “hair cortisol”).

The search string used in PubMed was as follows:

(“PE” OR “pre-eclampsia” OR “hypertensive disorders of pregnancy”) AND (cardiovascular OR endothelial OR vascular OR “arterial stiffness” OR hemodynamic OR “heart disease” OR “future risk”) AND (placenta OR spiral OR atherosis OR infarct* OR malperfusion OR histopathology) AND (biomarker* OR angiogenic OR sFlt OR PlGF OR microRNA OR metabolomic* OR “hair cortisol”).

No initial filters for study design were applied to allow inclusion of a broad range of evidence. However, only human studies and mechanistically relevant in vitro or animal studies were included if they offered insights into PE pathophysiology.

### 4.2. Inclusion and Exclusion Criteria

We included the following:

- Original research (clinical, basic science, and translational studies),
- Systematic reviews and meta-analyses,
- Clinical guidelines or consensus statements relevant to PE’s cardiovascular, placental, or biomarker aspects.

Excluded were the following:

- Case reports,
- Abstracts without full-text availability,
- Non-peer-reviewed literature,
- Studies published in languages other than English.
- In vitro or animal experiments.

### 4.3. Study Selection and Review Process

Titles and abstracts were screened for eligibility by two reviewers independently. Full-text articles were obtained and reviewed when abstracts indicated potential relevance or when eligibility was unclear. Discrepancies in inclusion were resolved through discussion, with a third reviewer consulted as needed. The final selection prioritized clinical applicability, mechanistic insights, and relevance to high-risk stratification, prevention, and monitoring of PE.

## 5. Conclusions

PE is the paradigm for the intersection of obstetric and cardiovascular medicine: an acute illness with late consequences [5,37,53,57]. Its pathogenesis stems from placental maladaptation, but its clinical syndrome is a maternal vasculopathy, immune response, and metabolic disorder [5,10,11,24,25]. The evidence conforms to the paradigm that PE is more than a transient pregnancy syndrome but a harbinger of subsequent cardiovascular disease [70,71,72].

An appreciation of the full spectrum of PE, ranging from its molecular origins to its lifetime impact, calls for reorientation in clinical approach and in public health perspective [5,6,75]. Prevention, early diagnosis, individualized treatment, and prolonged follow-up must be integrated into maternal care policy [63,64,69,78]. Lastly, the management of PE offers a unique opportunity to improve perinatal outcome as well as the cardiovascular fate of women worldwide [1,2,3,4,5,6].

## Data Availability

No new data were created or analyzed in this study. Data sharing is not applicable to this article as it is a narrative review based on previously published literature.

## Abbreviations

ACOG	American College of Obstetricians and Gynecologists
ADMA	Asymmetric Dimethylarginine
AI	Artificial Intelligence
AMA	Advanced Maternal Age
APS	Antiphospholipid Syndrome
ASPRE	Aspirin for Evidence-Based PE Prevention (trial)
AT1-AA	Angiotensin II Type 1 Receptor Autoantibody
AUC	Area Under the Curve
BMI	Body Mass Index
CD46	Cluster of Differentiation 46
CNS	Central Nervous System
CRAE	Central Retinal Artery Equivalent
CRH	Corticotropin-Releasing Hormone
CRVE	Central Retinal Venular Equivalent
CVD	Cardiovascular Disease
COX-1	Cyclooxygenase-1
DDAH	Dimethylarginine Dimethylaminohydrolase
DIC	Disseminated Intravascular Coagulation
eNOS	Endothelial Nitric Oxide Synthase
EOPE	Early-Onset PE
FGR	Fetal Growth Restriction
FMD	Flow-Mediated Dilation
FMF	Fetal Medicine Foundation
GPx	Glutathione Peroxidase
HELLP	Hemolysis, Elevated Liver enzymes, Low Platelet count
HPA	Hypothalamic–Pituitary–Adrenal
IL-6	Interleukin 6
INHBA	Inhibin Subunit Beta A
ISSHP	International Society for the Study of Hypertension in Pregnancy
IVF	In Vitro Fertilization
MAP	Mean Arterial Pressure
MDA	Malondialdehyde
MeSH	Medical Subject Headings
ML	Machine Learning
miRNA	MicroRNA
MRI	Magnetic Resonance Imaging
NET	Neutrophil Extracellular Trap
NGAL	Neutrophil Gelatinase-Associated Lipocalin
NO	Nitric Oxide
NOS3	Nitric Oxide Synthase 3
OCT-A	Optical Coherence Tomography Angiography
PAPP-A	Pregnancy-Associated Plasma Protein A
PCOS	Polycystic Ovary Syndrome
PE	PE
PIERS-ML	Preeclampsia Integrated Estimate of Risk with Machine Learning
PlGF	Placental Growth Factor
PP13	Placental Protein 13
PRES	Posterior Reversible Encephalopathy Syndrome
PWV	Pulse Wave Velocity
ROS	Reactive Oxygen Species
sEng	Soluble Endoglin
sFlt-1	Soluble fms-like Tyrosine Kinase-1
sNEP	Soluble Neprilysin
SLE	Systemic Lupus Erythematosus
SOD	Superoxide Dismutase
TGF-β	Transforming Growth Factor Beta
Th17	T-helper 17 Cells
TNF-α	Tumor Necrosis Factor Alpha
Treg	Regulatory T Cell
USPSTF	United States Preventive Services Task Force
VEGF	Vascular Endothelial Growth Factor
WHO	World Health Organization
